# Quantitative Differences in Rumen Epithelium Proteins in Lambs Fed Wheat, Perennial Wheat, or Perennial Wheat plus Lucerne

**DOI:** 10.3390/proteomes11030027

**Published:** 2023-09-20

**Authors:** Jude Jessie Bond, Gordon Refshauge, Matthew T. Newell, Benjamin W. B. Holman, David Wheeler, Serey Woodgate, Karthik S. Kamath, Richard C. Hayes

**Affiliations:** 1 NSW Department of Primary Industries, Extensive Livestock Industry Centre, Trevenna Rd, University of New England, Armidale, NSW 2351, Australia; 2 Cowra Agricultural Research and Advisory Station, NSW Department of Primary Industries, Cowra, NSW 2794, Australia; gordon.refshauge@dpi.nsw.gov.au (G.R.); matt.newell@dpi.nsw.gov.au (M.T.N.); richard.hayes@dpi.nsw.gov.au (R.C.H.); 3 Wagga Wagga Agricultural Institute, NSW Department of Primary Industries, Wagga Wagga, NSW 2650, Australia; benjamin.holman@dpi.nsw.gov.au; 4 NSW Department of Primary Industries, Orange Agricultural Institute, Orange, NSW 2800, Australia; dave.wheeler@dpi.nsw.gov.au; 5 Australian Proteome Analysis Facility (APAF), Macquarie University, North Ryde, NSW 2109, Australia

**Keywords:** nutrient, transport, membrane, rumen, epithelium

## Abstract

The value of crops such as perennial wheat (PW) for grain and grazing compared to conventional wheat (W), or the addition of lucerne to PW (PWL) is still being determined. This research sought to determine if these diets were associated with changes in the membranebound proteins that transport nutrients in the rumen epithelium (RE). Crossbred ewes (Poll Dorset × Merino) were fed W, PW, or PWL (50:50) fresh-cut forage *ad libitum* for 4 weeks. Average daily gain (ADG; *p* < 0.001) was highest in the W-fed lambs compared to the PW and PWL. Metabolisable energy intake (MEI) was higher in lambs fed W (*p* < 0.001) compared to PW and PWL. In pairwise comparisons of the PW and PWL diet group we found protein abundance was significantly (*p* < 0.05, FDR < 0.05, Benjamini *p* < 0.05) different in fatty acid metabolism, oxidative phosphorylation, and biosynthesis of cofactors pathways. There were not any differences in protein abundance related to nutrient transport or energy metabolism in the RE between W- vs. PW- and W- vs. PWL-fed lambs. However, in the PW- vs. PWL-fed lambs, there was a difference in the level of proteins regulating the metabolism of fatty acids and energy production in the mitochondria of the rumen epithelium.

## 1. Introduction

Dual-purpose cereal crops for grain and grazing present many advantages for mixed farming systems (livestock/cropping). These include filling winter feed gaps to meet livestock nutritional requirements, resting other pasture paddocks, increasing animal carrying capacity, and assisting with the management of weeds [1]. Perennial cereals are also anticipated to be developed as dual-purpose crops [2] and may offer additional benefits over annual cereal crops associated with reduced frequency of sowing, resulting in improved soil health and reduced inputs on cropping land [3]. Although still under development, perennial cereals are envisaged to be grown in biculture (or polyculture mixtures) such as with legumes as an ongoing source of biologically fixed nitrogen [4,5]. The potential benefits to livestock of grazing mixtures of cereals and legumes compared to pure cereal diets are not well understood.

Legumes grown in mixtures with cereal crops offer the potential to reduce fertiliser nitrogen (N) requirements of the crop [6] and provide adequate energy and protein nutritive requirements for livestock. Their inclusion has been shown to enhance the muscle concentrations of copper, iron, and phosphorus without impacting the sensory attributes of meat [7]. In addition, they have also been shown to alter the mineral balance when offered to pen-fed lambs in comparison to diets without legumes [8], but the changes at the rumen protein level associated with conventional wheat (W) compared to perennial wheat (PW) and the addition or omission of lucerne on rumen epithelium (RE) proteins is unknown.

The transport of major nutrients (carbohydrates, protein, lipids) across the rumen epithelium is dependent on the concentration and co-transport of sodium in particular. Monovalent (sodium; Na, potassium; K) and divalent (Calcium; Ca, Magnesium; Mg) cations are transported with nutrients along concentration gradients from the rumen fluid across the plasma membrane, and into the RE cells [9,10,11,12]. From here, nutrients enter metabolic pathways that provide energy for maintenance of cellular processes, with any excess being absorbed into the blood and distributed to tissue in the animal’s body. The primary sources of energy from microbial fermentation of plant fodder are short-chain fatty acids [13]. The main source is dietary, although mobilization from cellular stores can occur. Despite the widespread use of cereal crops (wheat, oats, triticale, barley) in conventional grazing systems, those diets are known to have a high ratio of K:Na [14]. This mineral imbalance has been associated with metabolic disorders in high-producing livestock, such as hypocalcaemia and hypomagnesaemia, but has also been implicated in lower growth rates in lambs, but the reason for this is not fully understood [15,16].

Using tandem MS with high levels of identification and recently developed proteomics procedure [17], protein profiles that represent those that transport nutrients through the cell membrane and organelles that control metabolic pathways regulating the fate of nutrients in the rumen epithelium were quantified. The research here examines the response of RE membrane proteins of sheep fed conventional W compared to PW or PWL using the proteomics approach.

## 2. Methods and Materials

The current study followed the Australian Code of Practice for the Use of Animals for Scientific Purposes [18]. The research was carried out in accordance with approval by the Animal Ethics Committee of the NSW Department of Primary Industries, Orange (ORA18/21/022).

### 2.1. Feeding Regime

Methods used for the feeding study are described by Newell et al. [19]. Forty-eight crossbred ewes (Poll Dorset × Merino) were fed fresh-cut forage *ad libitum* for 4 weeks (15 May 2019–10 June 2019). The 48 sheep were assigned to 4 different diet groups, with 12 sheep per diet treatment group, and each animal housed within an individual pen. Feed was cut using a sickle bar mower and provided *ad libitum* twice daily. Drinking water was always available. Three diets were selected for proteomic investigation: (1) conventional wheat variety (W; *Triticum aestivum cv. EGA Wedgetail*); (2) perennial wheat (PW; *T. aestivum x Thinopyrum ponticum breeding line 11955*); and (3) perennial wheat and lucerne (PWL; *Medicago sativa cv. Titan 9*; 50:50). Thirty-nine rumen samples collected and processed for proteomics in each diet included *n* = 10 W, *n* = 8 PW, *n* = 12 PWL with each individual able to be traced back to the original pen feed intake measurements.

### 2.2. Dietary Intake, Mineral Composition, and Growth

Samples of the feed were collected weekly throughout each phase of the experiment and analysed for chemical composition. The chemical composition of the feed on offer was analysed by the Feed Quality Service (Department of Primary Industries, Wagga Wagga) using the methods described by the Australian Fodder Industry Association [20] to determine neutral detergent fibre (NDF), crude protein (CP), dry matter digestibility (DMD), and metabolisable energy (ME). A further set of samples was used to determine K, Na, Mg, and Ca content of the forage [21]. Uneaten forage was collected once a day, immediately before feeding the morning ration, and sorted in the case of the PWL diet to calculate the refusals of cereal and lucerne components and total dry matter intake (DMI). Mineral indices were determined from the percentage of mineral in the dry matter (% DM) for K:Na ratio and dietary cation–anion difference (DCAD; meq/100 g) from dietary intake of W-, PW-, and PWL-fed lambs as described by Newell et al. [19]. Metabolisable energy intake (MEI) was derived from DMI multiplied by ME concentration in the diet (Equation (1)).

MEI = DMI × ME (i)(1)

i = forage component.

Liveweight (LWT, kg) is the mean liveweight recorded on day (D) D0, D7, D14, D21, and D28 of the feeding trial. Average daily gain (ADG, g/d) is calculated from the total change in LWT (D28 LWT − D0 LWT) ∗ 1000 = g) divided by 27 days (g/d).

### 2.3. Rumen Epithelium Isolation, Protein Extraction, and Labelling for Quantification

At the end of the experiment, all the lambs were slaughtered at a commercial Australian abattoir and samples of rumen wall from the ventral sac were collected, washed in phosphate-buffered saline to remove plant matter, and immediately frozen on dry ice.

**Figure 1:**
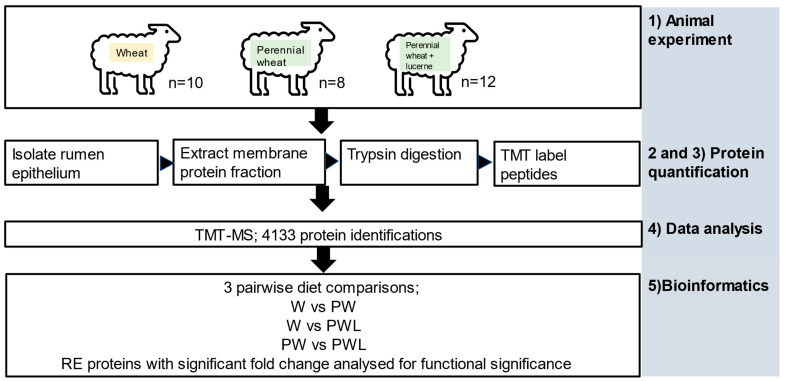
Workflow of the rumen epithelium (RE) proteome analysis. (1) Whole-depth rumen tissue from lambs fed three diets (wheat; W, perennial wheat; PW, perennial wheat and lucerne; PWL) were treated with enzyme and the epithelium isolated by microdissection. (2) A membrane fraction was prepared for each RE tissue sample. The extracts were then dialysed and SDS removed prior to trypsin digestion. (3) Each sample was labelled with 10-plex TMT reagents then separated by high pH (HpH) fractionation by HPLC. (4) Peptide high pH fractions were subjected to data-dependent acquisition–MS (DDA-MS) analysis of individual samples using TMT procedure for quantification. Protein identifications were made using Ensembl ovine database. (5) Three pairwise diet comparisons of RE proteins with significant fold change (FC) were analysed for functional significance using DAVID and KEGG.

The workflow diagram is shown in Figure 1.

The epithelium of each sample was removed enzymatically from underlying layers as described in Bond et al. [22]. Cell extracts were homogenised and fractionated into a membrane fraction using the MemPer Plus kit (Thermo Scientific, Rockford, IL, USA). Membrane protein extracts were dialysed against 1% SDS in 100 mM tris pH 8.5. Protein concentration was determined using the 2D quant Kit (GE Healthcare, Princetown, NJ, USA).

An equal amount of protein from each of the samples was processed for mass spectrometry analysis using S-Trap proteomics sample preparation kit, as per manufacturer’s instructions (Protifi, Huntington, NY, USA). Proteins were proteolytically digested with trypsin. Following the digestion, each sample (~100 μg) was labelled with 10-plex TMT reagents as per manufacturer’s instructions [23]. Samples were pooled as per pooling schema of 10-plex kit.

Each 10-plex TMT-labelled protein set was pre-fractionated using offline high pH (HpH) reversed phase high-performance liquid chromatography (HPLC) [23]. Peptide high pH fractions were subjected to data-dependent acquisition (DDA) on a Q-Exactive mass spectrometer (Thermo Scientific, USA) coupled to an UltiMate 3000 UHPLC (Thermo Scientific, USA). All the data files containing MS/MS spectra were searched against a reference database for the Ovine proteome (Oar v4. Ensembl, May 2017) using (Proteome Discoverer v 2.1.0.81, Thermo Scientific). Protein identifications were accepted with maximum of 2 missed cleavages trypsin, precursor mass tolerance 20ppm, fragment mass tolerance 0.02 Da, modifications of oxidation (M), deamidated (N, Q), Glu->PyroGlu, Gln->Pyro-Glu, acetyl (n-terminus) and TMT10plex (N-term) and carbamidomethyl (C), protein, peptide, and PSM FDR < 1%. Protein abundance values for each of the proteins across different treatment and control condition was exported into an Excel workbook.

Sodium-dodecyl sulfate (SDS) was removed to a suitable level from each sample so that it did not interfere with trypsin digestion and mass spectrometry procedures, by using S-Trap proteomics sample preparation kit (Protifi, Huntington, NY, USA). In brief, individual samples from each sheep and each fraction (~1 μg/fraction) were analysed on a TripleTOF 6600 mass spectrometer (SCIEX) as described previously by Kamath et al. [24]. TMT-labelled proteins were separated using offline high pH (HpH) reversed phase high-performance liquid chromatography (HPLC). Peptides were subjected to data-dependent acquisition (DDA) LC-MS/MS. All the MS/MS spectra were searched against a reference database for the Ovine proteome (Oar v4. Ensembl, May 2017) using (Proteome Discoverer v 2.1.0.81, Thermo Scientific). Protein identifications were accepted with maximum of 2 missed cleavages trypsin, precursor mass tolerance 20ppm, fragment mass tolerance 0.02 Da, modifications of oxidation (M), deamidated (N, Q), Glu->PyroGlu, Gln->Pyro-Glu, acetyl (n-terminus), and TMT10plex (N-term) and carbamidomethyl (C), protein, peptide, and PSM FDR < 1%.

Proteins were deemed differentially expressed with ANOVA *p*-value < 0.05, and the protein fold change (FC) > 1.2. The data files and search results are available at PRIDE data (https://www.ebi.ac.uk/pride/archive/, accessed on 8 August 2023, PRIDE ID: PXD044135) and Supplementary File S1.

### 2.4. Statistical and Bioinformatics Analysis

Significant differences in dietary intake of each sheep analysed for quantitative changes in protein abundance were analysed using Minitab (v18; www.minitab.com, accessed on 23 April 2023) [25]. The variables DMI (kg DM), MEI (MJ ME/kg DM), (% DMI) Na, K, Ca, Mg, K:Na ratio, dietary cation–anion difference (DCAD; meq/100 g DM), LWT (kg), and ADG (g/d) were analysed using a generalised liner model with diet (W, PW, or PWL) fitted as factor. Difference between diets was analysed using a post hoc Bonferroni test.

Differences in the abundance of rumen epithelium proteins across the 3 diets (*p* < 0.05 and fold change > 1.2) were analysed using ANOVA. Three pairwise comparisons of the mean intensity derived from the MS data for each protein were examined to find differences in protein abundance between two diets. *t*-tests were performed to compare the protein FC between 2 treatment groups. These were (1) W vs. PW, (2) W vs. PWL, and (3) PW vs. PWL. The FC is the mean intensity of a protein in one diet divided by another diet (e.g., FC W/PW). These FC values were converted to log 2 in Microsoft Excel. Significant differences in FC of proteins (FC > 1.2; *p* < 0.05) were subjected to further analysis for functional enrichment of biological processes. Firstly, all Ensembl protein accession numbers were converted to uniport KB accession numbers in www.uniprot.org using the retrieve mapping tool. To find functional enrichment of UniProt protein identifications for Kyoto Encyclopedia of Genes and Genomes (KEGG) pathways in each pairwise comparison the list of proteins with a significant FC >1.2, (*p* < 0.05) was submitted to the Database for Annotation, Visualization, and Integrated Discovery (DAVID v6.8; https://david.ncifcrf.gov/, accessed on 10 January 2023). *Ovis aries* was selected as a species and gene list in the functional annotation tool. The list of UniProt protein identifiers in each significantly enriched pathway or functional process (*p* < 0.05, FDR < 0.05, Benjamini *p* < 0.05) was listed.

Prediction of membrane protein topology was analysed using DeepTMHMM [26]. Subcellular prediction was based on top score given using WoLF PSORT (https://wolfpsort.hgc.jp/, accessed on 23 May 2023) [27].

## 3. Results

### 3.1. Dietary Intake of Energy, Minerals, and Growth

Dietary feed and mineral intakes as well as growth data are summarised in Table 1. Dry matter intake (DMI) was significantly (*p* < 0.001) lower on average for PW compared to W- and PWL-fed lambs, although CP % (*p* < 0.001) and NDF % (*p* < 0.001) intake was lowest in the PWL-fed lambs. Metabolisable energy intake (MEI) was higher (*p* < 0.001) in W- compared to PW- and PWL-fed lambs. There were significant differences (*p* < 0.001) between diets for Na (% DMI), K (% DMI), Ca (% DMI), Mg (% DMI), K:Na ratio, and DCAD (meq/100 g DM). Sodium levels, as fed, were below the 0.012% minimum recommended [28] and K intake was above the maximum recommended threshold level of 3% DM in all diets. The dietary intake of Ca was 2-fold higher than the minimum dietary recommendation of 4.8 g/d for a 50 kg lamb growing 250g/d [28] in PWL lambs, whereas the intake in the W- and PW-fed lambs was below the minimum threshold. Intake of Mg was similar and above the minimum threshold of 1.5 g/d [28] in all diets. The K:Na ratio of the diet was higher (*p* < 0.001) in the PW and lowest in the PWL. Each diet was at a higher level than found in forages with comparable ME for K:Na ratio [16]. The DCAD index was significantly (*p* < 0.001) different between diets and was lowered by 30% when lucerne was added to the PW diet. All diets had a DCAD higher than the recommended threshold (<12 meq/100 g DM) [14]. There were no significant differences in the final liveweights of lambs between all diets although ADG was on average 53% higher (*p* < 0.05) in the W-fed lambs compared to the PW and PWL.

### 3.2. Protein Identifications and Predicted Sub Cellular Location

**Figure 2:**
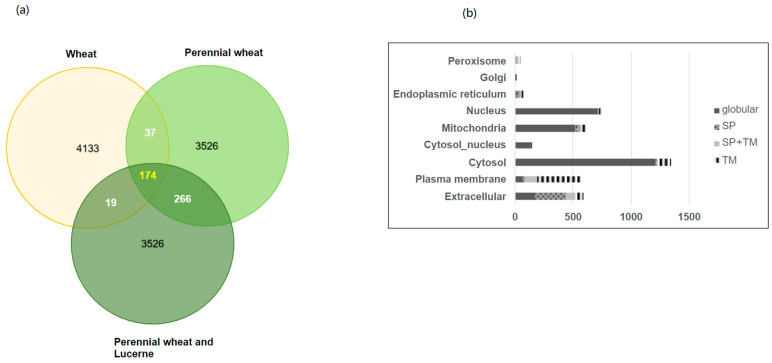
(**a**) Venn diagram of total number of rumen protein identifications (black text) in circles. Effect of diet on number of proteins with significant differences in protein abundance (FC > 1.2, *p* < 0.05) analysed by pairwise comparison is shown in the overlap of circles (white or yellow text). Number of proteins with significant differences between W, PW, and PWL was analysed by ANOVA. (**b**). Subcellular category (*y*-axis) and number of proteins in each category (*x*-axis) are represented in each bar. The proportion of proteins in each subcellular category predicted as globular (grey), with an N-terminal signal peptide (SP, textured grey), transmembrane domain (TM, black bands), or with SP + TM prediction (light grey) in the RE is shown.

The numbers of proteins identified in RE of lambs fed the three diets are shown in Figure 2a. In the PW and PWL diet groups, 3526 proteins, and in the W group 4133 proteins were identified. Subcellular location of proteins identified in the membrane fraction using WoLF PSORT predicted 1342 cytosol, 150 cytosol and nucleusi, 745 nucleus, 589 extracellular, 607 mitochondria, 574 plasma membrane, 72 endoplasmic reticuluma, 46 peroxisome, and 7 golgi proteins.

Of the proteins identified, 23% were predicted to be membrane proteins with at least one transmembrane domain (TM). The relative proportion of proteins predicted by DeepTMHMM as a globular (Glob), proteins with a signal peptide (SignalP), alpha-helical transmembrane prediction (TM), and transmembrane protein with signal peptide (SP + TM) are shown in the bar graph for the total in each subcellular location in Figure 2b.

### 3.3. Biological Function of Quantitative Differences in Protein Abundance with Diet

**Figure 3:**
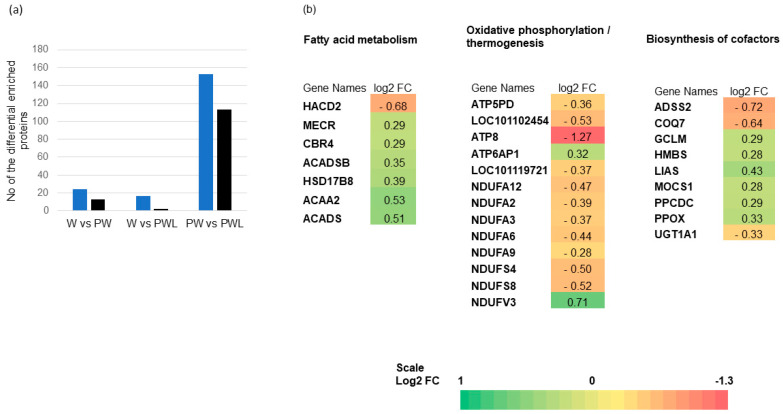
(**a**). Histogram of 3 pairwise comparisons of diets W vs. PW, W vs. PWL, and PW vs. PWL showing number of proteins with significant positive (blue) or negative (black) log2 fold change (FC). (**b**). Heat maps of significant functional pathways associated with differences in RE proteins log FC between lambs fed PW or PWL. The protein gene name and level of FC are colour-coded (see colour scale). Biological processes enriched include fatty acid (FA) metabolism, oxidative phosphorylation, and biosynthesis of cofactors.

The number of proteins with significant fold change (FC > 1.2, *p* < 0.05) in each pairwise comparison is shown in Figure 3a.

There was no significant enrichment of metabolic pathways in the comparison of W vs. PW and W vs. PWL diets. Nonetheless, the PW and PWL diet group had 153 proteins with a positive FC and 113 with a negative FC in protein abundance (Figure 3a). Of these, we found three functional pathways (KEGG) that were significantly (*p* < 0.05, FDR < 0.05, Benjamini *p* < 0.05) enriched. These were fatty acid metabolism, oxidative phosphorylation, and biosynthesis of cofactors. A summary of the pathway enriched for the PW vs. PWL diet comparison and the associated proteins log FC is shown in Figure 3b. In the fatty acid metabolism pathway, six of the proteins had a higher abundance in the PW- compared to the PWL-fed rumen epithelium, whereas 11/13 proteins in the oxidative phosphorylation/thermogenesis pathway had a higher abundance in the PWL- compared to the PW-fed lambs RE.

Although no enrichments of glycolysis or tricarboxylic acid cycle (TCA cycle) pathways were found using DAVID functional analysis, we quantified a significant difference for three glycolytic proteins (LOC101113460; FBP, ENO2, PDK4) and two tricarboxylic acid (TCA) cycle enzymes (ACO2, IDH3B) in the PW vs. PWL comparison (Table 2). Glycolysis is of relevance as it is a major cellular energy-generating pathway. PW-fed lambs RE were observed to have a higher protein serine/threonine kinase (PDK4) abundance than those fed the PWL. Similarly, aconitate hydratase (ACO2) which converts isocitrate from oxaloacetate and isocitrate dehydrogenase (NAD) and subunit beta (IDH3B) which converts isocitrate into alpha-ketoglutarate in the TCA cycle were both more abundant (FC > 1.2; *p* < 0.05) in the PW-fed lambs RE compared to those fed PWL.

### 3.4. Significant Differences in Abundance of Nutrient Transporters between Diets

A list of all transporters identified can be found in Supplementary File S2. Two main families of transporters were identified, solute carrier family (SLC; *n* = 69), and ATP binding cassette transporters (ABC; *n* = 15). Other included aquaporins, chloride ion channels, and water channels. We identified 180 transporters of which 102 had more than one predicted TMHMM indicating they are membrane proteins.

Of the families of SLC transporter proteins [30], 13 families (5, 6, 8, 9, 10, 13, 20, 23, 24, 28, 34, 38, 43) are involved in Na transport. Just two of these families, SLC5 (Na/glucose, SLC5A1) and SLC9 (Na/H, SLC9A2, SLC9A3, SLC9A3R1, SLC9A6), are Na co-transporters of which none had a difference in protein abundance between diets. Of the ATPases involved in co-transport via Na or K, three Na/K ATPase subunits were identified (ATP1A1, ATP1B1, LOC114117869). There was no difference (*p* > 0.05) in protein abundance between diets in any of these proteins. Also identified were four potassium transporters, KCNAB2, KCNK5, SLC12A4, and SLC12A6, and one magnesium transporter (MAGT1), none of which had significant differences in abundance between diets. Fifteen calcium transporters were identified: CACNA2D1, MCU, TSPAN13, TRPV2, TRPV4, ATPIA1, ATP2A2, ATP2A3, ATP2B1, ATP2B3, ATP2C1, SLC25A12, SLC25A13, SLC25A24, and SLC25A25. Only mitochondrial SLC25A25 (1.2 FC; *p* < 0.001) and plasma membrane calcium ATPase (ATP2B3; 1.6 FC; *p* < 0.001) were higher in the PW-fed lambs compared to the PWL RE. Mitochondrial calcium uniporter (MCU; 1.38 FC; *p* < 0.05) had a higher abundance in the RE of PWL- compared to PW-fed lambs.

**Figure 4:**
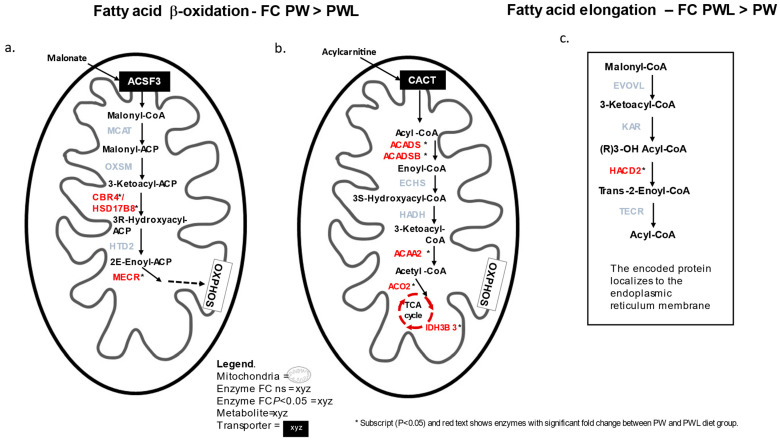
Fatty acid (FA) metabolic pathways describing the subcellular location and position in the process of FA degradation (β-oxidation) or synthesis (elongation). (**a**,**b**). Medium- or short-chain fatty acid β-oxidation metabolic pathways in the mitochondria showing enzymes (red text) that had FC PW > PWL (*p* < 0.05; black asterisk). (**c**). Very-long-chain fatty acid elongation enzyme HACD2 (red text) position in metabolic pathway which had higher FC PWL > PW (*p* > 0.05; black asterisk).

Two potential short-chain fatty acid (SCFA) transporters were identified in the RE of lambs fed the W, PW, and PWL diets: the SLC16A1 monocarboxylate transporter and maxi-anion transporter subunit protein SLCOA21. Neither had significant difference in abundance (FC > 1.2; *p* < 0.05) in the three diet treatments. Four FA transporters of solute-carrier family 27 members 1–4 (LACs) were identified but there were no significant differences in protein abundance between diets (Figure 4).

Four transporter proteins had significant FC (>1.2 FC and *p* < 0.05) in the PW compared to the PWL of which there was one plasma membrane iron transporter (SLC40A1). Two were located intracellularly in the mitochondria (SLC25A25, SLC25A32) [31], did not involve Na, K, or nutrient transport, and were higher in the PW compared to the PWL diet group.

## 4. Discussion

DMI was similar in lambs fed W and PWL and higher in those fed PW. However, ADG was highest in W-fed lambs, followed sequentially by those fed PW and PWL. Despite this, the MEI was around 15 MJ ME/kg DMI for all the diets, with a slightly higher MEI and CP% intake associated with the W diet compared to the PW and PWL diets. All diets fed provided a higher K:Na ratio than recommended [14]. The main difference in dietary mineral intake was a higher Ca content in the PWL diet compared to W and PW, attributable to higher concentrations of Ca in the lucerne fodder.

Several technical difficulties were overcome to firstly isolate RE proteins from underlying layers of muscle and connective tissue, then solubilise integral membrane proteins in a way that was suitable to quantify them using tandem MS with a high number of identifications using a recently developed proteomics procedure [17]. The procedure using TMT-MS to quantify membrane proteins provided a high number of protein identifications (4133) of which 574 were plasma membrane proteins and 180 were identified as nutrient transporters. Although no proteoforms are distinguished in the research, additional information including posttranslational modifications may be contained in the dataset and investigated in more detail in the future. There were no differences in the abundance of nutrient transporters in the RE of sheep fed diets of W compared to PW or PWL which all have a high K:Na ratio. The detailed coverage and quantification of the major nutrient transporters in the plasma membrane using the proteomic procedure presented provides strong evidence that the result is accurate. No functional biological processes were linked to quantitative changes in the protein abundance of RE in diet comparisons with W and PW or W and PWL. However, in PW- compared to the PWL-fed lambs, quantitative differences in RE proteins existed that were enriched for metabolic pathways fatty acid metabolism, oxidative phosphorylation, and biosynthesis of cofactors.

The passage of water-soluble minerals and nutrients across the impervious cell membrane (phospholipid bilayer) occurs through protein transporters. These include members of the solute carrier (SLC) and ATP-binding cassette (ABC) transporter families that are the predominant transporters found in RE [22]. Active transport occurs through transporters requiring ATP such as Na/K ATPases [32,33]. Other transporters are involved in fluid balance, such as volume-rectifying transporters (LREE) [34] and aquaporins, since the transport of ions, glucose, VFA, or AA is followed by water. Rumen fluid after feeding is associated with increased osmotic pressure (350–400 mosmol/L) [35,36]. Gemmel and Stacey [37] demonstrated that hyper-rumen osmotic pressure disrupts cell junctions in s. granulosum. In the present study we conclude that there were no major cellular disturbances due to quantitative differences in the multi-protein structures that make up cell junctions in the RE in any of the diets fed.

The transport of major nutrients such as glucose (SLC5) [38], amino acids (SLC15) [11], and volatile fatty acids (VFA; SLC16) [39,40] occurs when Na in exchange for hydrogen, Mg, Ca, and other nutrients create membrane potentials that are involved in nutrient transport through the epithelium cell layer to the blood. Despite the high K:Na ratio in the diet, there were no significant changes in the RE protein abundance of transporters required for major nutrients that involve Na in any diet fed. A similar result was found by Bond et al. [17] in the RE transporter proteins of sheep cereal and lucerne hay with a low or high methane emission phenotype. Also, the presence of the SLCOA21 protein subunit [41,42] suggests the existence of another mechanism that SCFAs could accumulate into the epithelium transcellularly and independently of Na.

According to Rogers and van’t Klooster [43], salivary Na is responsible for 95% of the total Na entering the rumen. Although the dietary intake of Na was low, it is most likely that salivary Na composed of extracellular reserves of sodium in bodily fluids provided adequate Na in the rumen fluid and there was no disturbance or change of abundance of Na transporter proteins detected. Observations in sheep fed these diets with a high K:Na ratio resulted in a decrease in plasma Na and Mg as well as decreases in the urinary excretion of Na and Mg (*p* < 0.001) over the 4-week study, with no difference between diets [8]. These findings illustrate there are several mechanisms available for the animal to maintain ion homeostasis despite an apparent lack of dietary Na in the cereal diets offered. In contrast, Dove and Kelman [15] observed between 36 and 42 g/d higher weight gain in lambs when a dual-purpose wheat diet was supplemented with NaCl (salt). The difference in ADG between the W- compared to PW- and PWL-fed lambs was a similar range (43–45 g/d). However, the level of Na in all diets was very low, and therefore, lucerne did not provide a strong enough contrast in Na concentration between diets to observe the effect of higher sodium levels on liveweight gain. Future studies may want to test pasture species that are known to have higher concentrations of Na than lucerne [44] or consider an experiment where NaCl (salt) is added as a supplement.

The Ca concentration of lucerne herbage was much higher than either of the cereals and as expected, intakes of Ca and Mg were higher in the PWL diet compared to PW or W diets. Research by Bhanugopan et al. [29] found that high dietary K resulted in impaired Ca absorption in the rumen, but not the abomasum. The present study suggests the rumen responded to a lower dietary Ca level and the plasma Ca in the PW diet [8] compared to the PWL diet with an increase in abundance of plasma membrane ATPase Ca transporter (ATP2B3) protein. The significant change of abundance in the SLC25A25 and MCU transporter is not relevant to mineral balance in the lambs’ bodily fluids since they occur in the membranes of the mitochondria and carry out specialised functions within the cell and in this organelle.

The significant enrichment for the KEGG pathway short-chain fatty acid degradation in PW-fed lambs compared to the PWL is an important finding. Enzymes ACADS, ACADSB, and ACAA2 had higher abundance in the PW than PWL. Each is an enzyme involved in fatty acid b-oxidation that occurs in the mitochondria. ACADS is an enzyme with an affinity for medium- or short-chain fatty acids, whereas ACADSB prefers the branched-chain acyl-CoA or 2-methylbutryl-CoA, which is a product of catabolism of isoleucine. In addition, 3-oxoacyl-[acyl-carrier-protein] reductase (CBR4) and (3R)-3-hydroxyacyl-CoA dehydrogenase (HSD17B8) as well as Enoyl-[acyl-carrier-protein] reductase (MECR) was found to have higher abundance in the PW-fed lambs compared to the PWL. CBR4 and HSD17B8 form a dimer to function and provide intermediate metabolites for the MECR enzyme in the breakdown of malonate in the mitochondria and participate in fatty acid oxidation. In contrast, in the cluster of enzymes found enriched for fatty acid metabolism, very-long-chain (3R)-3-hydroxyacyl-CoA dehydratase 2 (HACD2) had a higher FC in the PWL- compared to the PW-fed lambs and is involved in FA elongation. Located in the endoplasmic reticulum membrane HACD2 catalyses the third step in the conversion of long-chain fatty acids to very-long-chain fatty acids. Overall, the enzymes participating in FA degradation are specific for short to medium-chain or branched-chain fatty acids which would provide cellular energy to the RE in the PW- compared to the PWL-fed lambs.

In the glycolytic pathway, PW-fed RE protein serine/threonine kinase (PDK4) was higher in abundance than in the PW-fed. The PDK4 enzyme serves to regulate substrate decision-making by manipulating the pyruvate dehydrogenase (PDH) complex by encouraging the supply of acetyl CoA to come from the b-oxidation of fat rather than the combustion of glucose. Acetyl-CoA is then used in the TCA acid cycle and provides the substrates for the electron transport chain to generate ATP. Therefore, the key enzymes regulating glycolysis were probably instrumental in determining a preference for energy formation through β-oxidation of fatty acids rather than glycolysis in the RE proteins of PW-fed lambs compared to the PWL.

In contrast, in the PWL- compared to the PW-fed lambs there was a significant enrichment of proteins involved in the oxidative phosphorylation pathway of the mitochondria which provides energy in the form of ATP from b-oxidation of FAs or glycolysis through the breakdown of simple sugars (glucose) from complex carbohydrates found in fodder such as PW and PWL diets.

## 5. Conclusions

Despite the high K:Na ratio in all diets, there was no evidence that lambs fed either of the three diets had metabolic disturbances at the cell level in the RE. Also given the decrease in Na in the blood and urine reported in a supporting study on the same experiment [8], the research supports a conclusion that ion homeostasis in the rumen fluid was maintained by salivary Na and other extracellular fluids despite the apparent lack of dietary Na in the cereal or PWL diets. The quantitative differences in RE proteins found in the comparison of the PW- vs. PWL-fed lambs were related to cellular energy metabolism and appear to be independent of the mineral composition in the W, PW, and PWL diets.

## Figures and Tables

**Table 1:** Lambs were fed 3 diets; wheat (W), perennial wheat (PW), and perennial wheat and lucerne (PWL). Adjusted mean and standard error of mean (SEM), of dry matter intake (DMI), metabolisable energy intake (MEI), crude protein (% DMI), neutral detergent fibre (NDF% DMI), dietary concentration (% DMI) of calcium (Ca), sodium (Na), potassium (K), and dietary cation–anion difference (DCAD) adjusted for intake over the 28 days of the experiment. Minimum dietary requirement is stated in the adjacent column. Also, mean liveweight (LWT) and average daily gain (ADG) in weight over 27 days of measurement are included.

	W *n* = 10	PW *n* = 8	PWL *n* = 12	SEM ^1^	*p*-Value	Minimum Dietary Requirement ^2^
DMI (kg)	1.28 ^a^	1.21 ^b^	1.32 ^a^	0.015	0.001	na
MEI (MJ ME/kg DM)	15.70 ^a^	14.58 ^b^	15.1 ^b^	0.184	0.001	na
CP (%) intake	33.53 ^a^	29.48 ^b^	25.04 ^c^	0.355	0.001	na
NDF %	53.56 ^a^	54.39 ^a^	45.29 ^b^	0.436	0.001	na
Na (%)	0.009 ^a^	0.005 ^b^	0.012 ^c^	0.0003	0.001	0.07–0.12%
K (%)	4.24 ^a^	4.70 ^b^	3.47 ^c^	0.032	0.001	0.5%
Ca (%)	0.31 ^a^	0.34 ^a^	0.82 ^b^	0.006	0.001	0.3–0.5%
Mg (%)	0.12 ^a^	0.12 ^a^	0.14 ^b^	0.001	0.001	0.09–0.2%
K:Na	524 ^a^	937 ^b^	313 ^c^	0.081	0.001	6–7 ^‡^
DCAD (meq/100 g DM)	67.5 ^a^	77.9 ^b^	53.3 ^c^	0.63	0.001	<12–35 ^†^
Liveweight (kg)	47.8	47.4	47.6	0.62	ns	na
ADG (g/d)	127.4 ^a^	84.3 ^b^	82.2 ^b^	36.80	0.02	na

Week of measurement was fitted as a covariate for intake data and was significant *p* < 0.05 in all mineral or ratios presented. ^1^ Average of values of standard error of the mean (SEM) for diet within each comparison. Different superscripts are shown where there is a significant difference between diet ^a–c^ (*p* < 0.05). Abbreviations. na; not applicable. ^2^ Unless stated cited from [28,29]. ^‡^ Cited from [16], ^†^ [14].

**Table 2:** A summary of quantitative differences in enzymes involved in glycolysis and fatty acid metabolism pathways found to be significantly higher inrumen epithelium (RE) of lambs fed PW compared to PWL. Columns represent metabolic pathway, UniProt accession number, protein name, gene name, fold change (FC), and *p*-value. Further details of the identifications are found in Supplementary File S3.

Pathway	UniProt Accession	Protein Name	Gene Name	FC	*p*-Value
Gluconeogenesis					
	O18751	Glycogen phosphorylase, muscle form (EC 2.4.1.1) (Myophosphorylase)	PYGM	1.38	0.006
	W5PFT7	Fructose-bisphosphatase 2	FBP2	1.37	0.002
Glycolysis					
	W5P5C0	Enolase 2	ENO2	1.51	0.01
	W5NZZ4	Protein serine/threonine kinase (EC 2.7.11.)	PDK4	1.55	0.05
Methylglyoxal shunt	W5Q540	Hydroxyacylglutathione hydrolase	HAGH	1.20	0.005
TCA cycle	W5QAA9	Aconitate hydratase, mitochondrial (Aconitase) (EC 4.2.1.)	ACO2	1.22	0.02
		Isocitrate dehydrogenase [NAD] subunit B	IDH3B		
Fatty acid degradation					
	W5PUC2	Acyl-CoA dehydrogenase short chain	ACADS	1.42	0.005
	W5PHF2	Acyl-CoA dehydrogenase short/branched chain	ACADSB	1.27	0.01
	W5PW04	Acyl-CoA dehydrogenase family member 8	ACAD8	1.23	0.02
Fatty acid degradation					
	W5PYE3	Lipase	LIPN	1.50	0.004 PWL>
	W5PT19	Phospholipid-transporting ATPase	ATP8B3	1.61	0.003
	W5P1M4	Acetyl-CoA acyltransferase 2	ACAA2	1.44	0.014
	W5Q9G8	Enoyl-CoA delta isomerase 1	ECI1	1.23	0.008
	W5NWE0	Mitochondrial trans-2-enoyl-CoA reductase	MECR	1.22	0.02
Fatty acid biosynthesis					
	W5QH76	Elongation of very-long-chain fatty acids protein 1 (EC 2.3.1.199) (very-long-chain 3-ketoacyl-CoA synthase 1)	ELOVL1	1.36	ns
	W5QG36	Very-long-chain (3R)-3-hydroxyacyl-CoA dehydratase (EC 4.2.1.134)	HACD2	1.58	0.02 PWL>
	W5PG98	Hydroxysteroid 17-beta dehydrogenase 8	HSD17B8	1.31	0.03 PW>
	W5QHK3	Methylcrotonoyl-CoA carboxylase 1	MCCC1	1.22	ns
	W5NTN7	Patatin-like phospholipase domain containing 6	PNPLA6	1.20	ns

## Data Availability

The datasets used and/or analysed during the current study are available from the corresponding author on reasonable request. All data generated or analysed during this study are included in this published article and its supplementary information file.

## Supplementary Materials

The following supporting information can be downloaded at: https://www.mdpi.com/article/10.3390/proteomes11030027/s1, File S1: Dietary intake and liveweight data; File S2: Protein subcellular location and predicted category; File S3: Quantitative differences in protein abundance between diets.
